# Editorial: Natural compounds from plant: microbiome-targeted therapeutic strategy for gastrointestinal disorders

**DOI:** 10.3389/fcimb.2025.1736556

**Published:** 2025-12-11

**Authors:** Wei Chen

**Affiliations:** Department of Gastroenterology, Shanghai Sixth People’s Hospital Affiliated to Shanghai Jiao Tong University School of Medicine, Shanghai, China

**Keywords:** gut microbiome, natural compounds, gastrointestinal disorders, inflammation, host-microbe interactions

The intricate ecosystem of the human gut microbiome stands as a pivotal interface between host health and disease, particularly in the context of gastrointestinal (GI) disorders. The burgeoning field of microbiome research has illuminated the profound influence of microbial communities on inflammation, immune homeostasis, and metabolic pathways within the gut and beyond. Concurrently, there is a resurgent interest in natural compounds derived from plants, which have been used for centuries in traditional medicine, for their multi-targeted therapeutic potential. This Research Topic, *“Natural Compounds from Plant: Microbiome-targeted Therapeutic Strategy for Gastrointestinal Disorders,”* was conceived to explore the synergistic potential of these two frontiers. The collected articles within this topic provide compelling evidence that plant-derived natural compounds can exert significant therapeutic effects by precisely remodeling the gut microbiome and modulating associated host signaling pathways, offering novel strategies for managing a spectrum of GI and related disorders.

The contributions herein span a remarkable range of conditions, from localized intestinal inflammation to systemic diseases linked via the gut-liver and gut-brain axes. A prominent theme is the amelioration of inflammatory bowel disease (IBD). The study by Chen et al. demonstrates that Qing-Re-Hua-Shi Decoction (QRHSD) alleviates DSS-induced colitis through a multi-pronged mechanism. It not only restores gut microbial balance by enriching *Lactobacillus* and reducing *Morganella* but also rectifies metabolic disturbances and concurrently suppresses key pro-inflammatory pathways, including PI3K/AKT, NF-κB, and MAPK. This holistic, systems-level approach exemplifies how complex botanical formulations can integrate microbiome and host-directed actions. Similarly, coumarin derivatives (Jung et al.) exhibit potent antibacterial activity, effectively reducing pathogen load in an infectious colitis model. This direct antimicrobial action prevents pathogenic expansion (e.g., *Enterobacteriaceae*) and subsequent inflammatory Th1/Th17 cell recruitment, showcasing a targeted strategy to correct dysbiosis-driven inflammation.

Beyond the gut itself, the influence of microbiome-targeted interventions on remote organs is a critical area of investigation. The work on physcion for alcoholic liver fibrosis (ALF) (Bai et al.) elegantly connects gut microbial changes to hepatic pathophysiology via the gut-liver axis. Physcion’s hepato-protective effect is linked to the inhibition of the HMGB1/NLRP3 inflammasome pathway and pyroptosis, a pro-inflammatory cell death. Crucially, this was associated with a restoration of a healthier gut microbial landscape, underscoring the microbiome’s role as a driver of liver disease. Extending this concept to the central nervous system, the review on pycnogenol (PYC) (Chen et al.) proposes a therapeutic model for neurodevelopmental disorders (NDDs) like ASD and ADHD. It posits that PYC can modulate the gut-brain axis by increasing beneficial microbes such as *Akkermansia muciniphila* and butyrate-producing bacteria, thereby influencing microbial metabolites and dampening neuroinflammation via NF-κB and MAPK pathways. This highlights the potential of natural compounds to address complex disorders rooted in gut-brain communication.

The pharmacological validation of traditional remedies is another cornerstone of this topic. The investigation into *Cymbopogon proximus* essential oil (EOCP) (Althurwi et al.) provides a robust scientific basis for its traditional use in treating hyperactive GI disorders like diarrhea and spasms. The study reveals that EOCP acts as a dual inhibitor of muscarinic receptors and calcium channels, directly relaxing intestinal smooth muscle. This mechanism-focused research bridges traditional knowledge with modern pharmacodynamics. In a critical care context, Shenfu Injection (SFI) (Li et al.) demonstrates efficacy in a sepsis model by simultaneously curbing systemic inflammation and reshaping the gut microbiome, notably reducing pathogenic *Escherichia-Shigella* and promoting *Akkermansia* and *Lactobacillus*. This positions SFI as a promising candidate for modulating the dysregulated host-microbe interface in sepsis.

Finally, the study on Juemingzi (Senna seed extracts) (Narrowe et al.) introduces the concept of “targeted remodeling” of the human gut microbiome. Using an advanced *in vitro* fermentation model, it reveals that the extract exerts strong, taxon-specific antimicrobial effects, nearly eliminating *Bacteroidota*. This precise manipulation, while altering fermentative outputs, opens avenues for using such compounds as tools for directed microbial community engineering, albeit with a need for careful dosage and adjunct prebiotic strategies.

Collectively, the research presented in this Topic paints a cohesive picture: plant-derived natural compounds are powerful modulators of the gut microbiome, and their therapeutic efficacy is often mediated through this microbial restructuring. The mechanisms are diverse, encompassing direct antimicrobial activity, enrichment of beneficial probiotics, suppression of core inflammatory cascades (NF-κB, MAPK, PI3K/AKT, NLRP3), and restoration of gut barrier and organ function. These findings firmly place microbiome-targeted strategies at the forefront of a new paradigm for treating GI disorders. They argue for a future where therapies are not merely anti-inflammatory or antibiotic but are designed to restore the entire ecological and signaling network of the gut. We hope this Research Topic of work inspires further interdisciplinary research to unravel the complex dialogues between plant phytochemistry, gut microbes, and host physiology, ultimately accelerating the development of effective, natural product-based microbiome therapeutics.

